# Dextran Methacrylate Reactions with Hydroxyl Radicals and Hydrated Electrons in Water: A Kinetic Study Using Pulse Radiolysis

**DOI:** 10.3390/molecules28104231

**Published:** 2023-05-22

**Authors:** Kamila J. Szafulera, Radosław A. Wach, Piotr Ulański

**Affiliations:** Institute of Applied Radiation Chemistry, Faculty of Chemistry, Lodz University of Technology, Wroblewskiego 15, 93-590 Lodz, Poland; kamila.szafulera@gmail.com (K.J.S.); piotr.ulanski@p.lodz.pl (P.U.)

**Keywords:** dextran methacrylate, pulse radiolysis, radiolysis of polysaccharides, cross-linking, reaction kinetics, macroradicals, ionizing radiation

## Abstract

Dextran methacrylate (Dex-MA) is a biodegradable polysaccharide derivative that can be cross-linked by ionizing radiation. It is therefore considered a potential replacement for synthetic hydrophilic polymers in current radiation technologies used for synthesizing hydrophilic cross-linked polymer structures such as hydrogels, mainly for medical applications. This work is focused on the initial steps of radiation-induced cross-linking polymerization of Dex-MA in water. Rate constants of two major transient water radiolysis products—hydroxyl radicals (^•^OH) and hydrated electrons ($e_{\mathrm{aq}}^{-}$)—with various samples of Dex-MA (based on 6–500 kDa dextrans of molar degree of substitution or DS with methacrylate groups up to 0.66) as well as non-substituted dextran were determined by pulse radiolysis with spectrophotometric detection. It has been demonstrated that these rate constants depend on both the molecular weight and DS; reasons for these effects are discussed and reaction mechanisms are proposed. Selected spectral data of the transient species formed by ^•^OH- and $e_{\mathrm{aq}}^{-}$-induced reactions are used to support the discussion. The kinetic data obtained in this work and their interpretation are expected to be useful for controlled synthesis of polysaccharide-based hydrogels and nanogels of predefined structure and properties.

## 1. Introduction

Ionizing radiation has been long recognized as a useful tool for cross-linking polymers [1,2,3]. Numerous large-scale technologies based on this idea have been developed and continue to be used in industrial practice [4]. Versatility of radiation technology allows us to perform cross-linking not only in the solid state but also in solution. In particular, irradiation of hydrophilic polymers in water is a simple way to synthesize hydrogels. Here, cross-linking (gel formation) can be combined in one technological step with sterilization, making this approach particularly suitable for synthesizing hydrogel-based biomaterials and medical products, such as hydrogel wound dressings [5,6,7]. While this technology has been employed for many years and the products—based on radiation-cross-linked synthetic polymers of excellent biocompatibility—have a well-established position on the market, emerging trends and customer expectations stimulate new developments in this field. One of them is focused on replacing synthetic polymers with those based on natural and renewable resources. While successful attempts have been made in cross-linking various biobased compound and structures, including proteins [8], one of the main research trends, stimulated by potential applications, is to seek solutions allowing the use of ionizing radiation to synthesize cross-linked structures based on polysaccharides.

Research on the influence of ionizing radiation on polysaccharides has been conducted since the 1950s [9,10,11]. Basically, polysaccharides are known as polymers that undergo mainly degradation upon exposure to ionizing radiation. Reduction in molecular weight is predominantly caused by the scission of glycosidic bonds present between repeating units. Degradation occurs both when irradiation is performed in the solid state and in the aqueous solution [12,13,14,15]. This phenomenon is industrially exploited in order, for instance, to improve solubility or control-reduce molecular weight for specific applications [15]. Nevertheless, a cross-linking reaction may be present or even predominate over scission of polymer chains during exposure to ionizing radiation. Under certain conditions of irradiation and in the presence of additives—participating or mediating in the cross-linking, molecular weight of the polysaccharide can increase, moreover an insoluble fraction—the gel—can be formed [16,17]. Efficient cross-linking of polysaccharides by ionizing radiation without cross-linking agents was first demonstrated by Yoshii and coworkers, who described radiation-induced cross-linking of cellulose derivatives such as hydroxypropyl or carboxymethyl cellulose (CMC) processed in highly concentrated aqueous solutions, resulting in stable hydrogels with covalent bonds linking polysaccharide chains [18,19]. While handling and deoxygenating highly viscous, concentrated polysaccharide solutions may be difficult, it has been subsequently proved that CMC can be radiation-cross-linked or even in dilute solutions when certain conditions are met [20]. Other examples demonstrate additive-free radiation cross-linking of chitin and chitosan derivatives [21,22], leading to the formation of materials to be used in the field of regenerative medicine [23,24,25,26].

In general, cross-linked polysaccharides seem to be a perfect starting material for the design of numerous future applications in the field of biomaterials. Many polysaccharides have excellent biocompatibility, are non-toxic and biodegradable. One of them is dextran—a bacterially derived homopolysaccharide, included in the WHO Model List of Essential Medicines, with a long-standing record of medical applications. Dextran contains a large amount of hydroxyl group present in sugar residual, where hydrogen atoms can be replaced by numerous substituents. This approach allows the introduction different functional moieties into chemical structure, yielding dextran derivatives with specific properties, which can be further engineered to design and obtain a variety of microstructural forms such as fibers, scaffolds or hydrogels, with potential application in the field of tissue regeneration [27,28].

In our recent study, we focused on dextran methacrylate (Dex-MA), which, due to the presence of a polymerizable methacrylic group (-MA), is capable of forming biocompatible, insoluble macroscopic hydrogels at low doses of ionizing radiation [29]. Another successful application of radiation for cross-linking of dextran methacrylate and hyaluronan methacrylate cryogels has been demonstrated by Reichelt and coworkers [30,31,32,33]. Therefore, radiation technology seems to be a versatile, additive-free and clean tool to produce chemically cross-linked, biocompatible hydrogels based on methacrylated polysaccharides.

**Figure 1:**
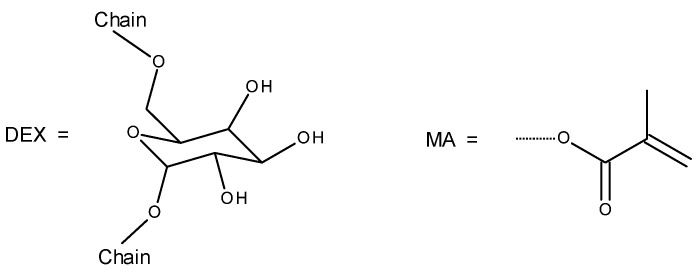
Structural components of the studied polymers: dextran (Dex) and methacrylate (MA) moiety.

The structure of Dex and the methacrylate moieties are shown in Figure 1.

The present report describes basic studies on radiolysis of dextrans (Dex) and dextran methacrylates with various degree of substitution (DS) with MA moieties and a wide range of initial molecular weight of the parent polysaccharide in aqueous solutions. These studies were performed by pulse radiolysis with spectrophotometric detection, using both a direct approach and competition kinetics. The focus of this work is the kinetics of the first step of Dex-MA radiolysis in water, namely, its reactions with the main transient products of water radiolysis, i.e., hydroxyl radicals and hydrated electrons. We aimed at determining the dependencies of the respective rate constants on the average molecular weight and degree of substitution (including non-substituted substrates), and thus identifying the role played by the MA moiety in the overall reactivity of Dex-MA under irradiation and formulating the general mechanism of radiation-induced reactions in aqueous Dex-MA solutions, in the context of the following polymerization and cross-linking steps. We hope that these results will be helpful for the rational design of radiation synthesis of new cross-linked polysaccharide-based products, particularly hydrogels and nanogels for medical applications.

## 2. Results

### 2.1. Generation of Hydroxyl Radicals and Hydrated Electrons and Their Reactivity towards Polysaccharides in Dilute Aqueous Solutions

Essentially, in the radiolysis of dilute aqueous solutions of a polysaccharide, polymer-derived radicals are formed as a consequence of reaction with initial products of water radiolysis. When the aqueous solution of polymer is irradiated, most of the supplied energy is absorbed by water molecules leading to the formation of wide range of very reactive water-derived radicals, as follows [13,34,35,36,37].

$$H_{2}O\;\to \;{}^{•}\mathrm{OH}+{\;e}_{\mathrm{aq}}^{-}+{\;H}^{\cdot }+{\;H}^{+}+H_{2\;}+{\;H}_{2}O_{2}$$

The main products are hydroxyl radicals, hydrated electrons and hydrogen atoms, which, in typical conditions of gamma-ray or electron beam irradiation, are formed with radiation-chemical yields of 0.28, 0.28 and 0.06 μmol/J, respectively. Hydrated electrons are relatively unreactive towards unsubstituted sugars and polysaccharides containing no carbonyl bonds. This usually manifests itself by the fact that the decay rate of hydrated electrons in water is not significantly accelerated by addition of these substrates. In contrast, hydroxyl radicals are known to react with sugars at a considerably high rate constant (e.g., for ^•^OH reaction with glucose *k* = 1.5 × 10^9^ dm^3^ mol^−1^ s^−1^ and with saccharose *k* = 2.3 × 10^9^ dm^3^ mol^−1^ s^−1^ [35]) by abstracting hydrogens from carbon atoms, thus leading to the formation of carbon-centered radicals. Selectivity of ^•^OH attack is usually low. H atoms participate in analogous reactions, albeit typically at a lower rate, and since their yield is also much lower than that of ^•^OH, H-atom reactions are often neglected in kinetic and product analysis, at least when irradiation is performed in solutions of neutral pH. Carbon-centered radicals resulting from ^•^OH attack in the absence of oxygen undergo transformations leading mostly to scission of glycosidic bonds (and thus to degradation and reduction of molecular weight). More detailed description of radiation-induced reactions in typical non-modified polysaccharides can be found elsewhere [12,13,38].

Cross-linking of polysaccharides under irradiation (in the absence of such additives as cross-linking agents or synthetic polymers) requires the presence of at least a moderate number of particular functional groups capable of promoting radical recombination or propagation. In carboxymethylcellulose and other carboxymethylated polysaccharides, this role is played by the methylene group; radicals located there can undergo cross-linking [20,21,22,23,24,39,40]. In methacrylated hyaluronate or dextran, methacrylate groups are expected to undergo polymerization when suitable initiation is provided by irradiation.

Our recent study indicates that irradiation of aqueous solutions of Dex-MA results in very efficient hydrogel formation at low irradiation doses [29]. This is expected to be both due to efficient initiation and propagation steps. In the following sections, the kinetics of initiation (formation of Dex-MA radicals) are studied in some detail, in order to determine whether and how two main products of water radiolysis, ^•^OH and $e_{\mathrm{aq}}^{-}$, react with the methacrylated dextran derivatives and what the influence of initial molecular weight of Dex and DS of Dex-MA on the rate constant of these reactions is.

### 2.2. Reactivity of Hydroxyl Radicals with Dextran and Dextran Methacrylate

In principle, it is possible to determine the rate constant of reaction of hydroxyl radicals with a solute in water by following the decay of ^•^OH radical-derived absorption or by following the increase in absorbance of the reaction product at a characteristic wavelength. However, due to a very low extinction coefficient (ε = 575 dm^3^ mol^−1^ cm^−1^, λ_max_ = 230 nm) of hydroxyl radical and spectral overlap with other chemical compounds in the UV region [41], direct observation of hydroxyl radicals species is difficult and impractical. Moreover, absorption spectra of polysaccharide-derived carbon-centered radicals fall in the UV region (thus partially overlapping with ^•^OH), are broad and of low intensity (see e.g., [42] and Section 3 below). Therefore, we decided to apply the competition method, using thiocyanate as the competing ^•^OH scavenger. Hydroxyl radical reacts with SCN^−^ to generate (SCN)_2_^•−^ (*k* = 1.1 × 10^10^ dm^3^ mol^−1^ s^−1^ [35]), which is easy for spectrophotometric detection due to its high molar absorption coefficient at its maximum absorbance at 480 nm (reactions (1)–(2)) [43,44]. In the current experiments, the concentration of scavenger was fixed at 2 mM, whereas polymer concentration was varied. Aqueous solutions of polymers were prepared in advance to ensure their complete dissolution in water. All solutions were deaerated by saturation with N_2_O for at least 20 min immediately prior to irradiation. Saturation with nitrous oxide allowed us to scavenge the hydrated electrons (unwanted when studying ^•^OH reaction kinetics) and doubles the yield of hydroxyl radicals (reaction (3)).

(1) $${}^{•}\mathrm{OH}+{\;\mathrm{SCN}}^{-}\;\to {\;\mathrm{OH}}^{-}+{\;\mathrm{SCN}}^{\cdot }$$

(2) $${\;\mathrm{SCN}}^{\cdot }+{\;\mathrm{SCN}}^{-}\;\to {\left(\mathrm{SCN}\right)}_{2}^{•-}$$

(3) $$e_{\mathrm{aq}}^{-}+{\;N}_{2}O\overset{H_{2}O}{\to }\;{}^{•}\mathrm{OH}+{\mathrm{OH}}^{-}+{\;N}_{2}\;$$

**Figure 2:**
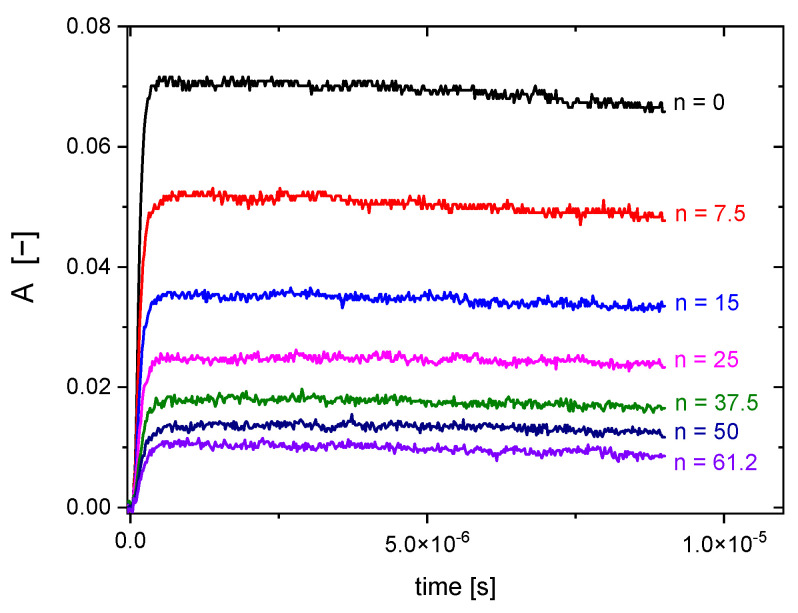
Pulse radiolysis of aqueous N_2_O-saturated solutions of 2 mM KSCN and various concentrations of Dex25-MA0.05, where *n* = [Dex25-MA0.05]/[SCN^−^]. Determination of rate constant of ^•^OH radical reaction with Dex25-MA0.05 by competition method with SCN^−^ as the competing scavenger. Kinetic traces at 480 nm (pulse duration 7 ns, dose per pulse 15 Gy).

For each polymer concentration, absorbance of (SCN)_2_^•−^ at 480 nm after the electron pulse was recorded. An exemplary set of kinetic traces for various polymer concentrations is shown in Figure 2.

Based on measurements of the maximum (SCN)_2_^•−^ absorbance, *A*_1_, as a function of polymer concentration ([polymer]), while knowing the absorbance in the absence of polymer, *A*_0_, and the rate constant of ^•^OH + SCN^−^ reaction (*k*_OH+scavenger_), the rate constants of the ^•^OH reaction with the studied polymer were calculated using Equation (4) [13,45].

(4) $$\frac{A_{0}}{A_{1}}-1=\frac{k_{\mathrm{OH}+\mathrm{polymer}}\cdot \left[\mathrm{polymer}\right]}{k_{\mathrm{OH}+\mathrm{scavenger}}\cdot \left[\mathrm{scavenger}\right]}$$

**Figure 3:**
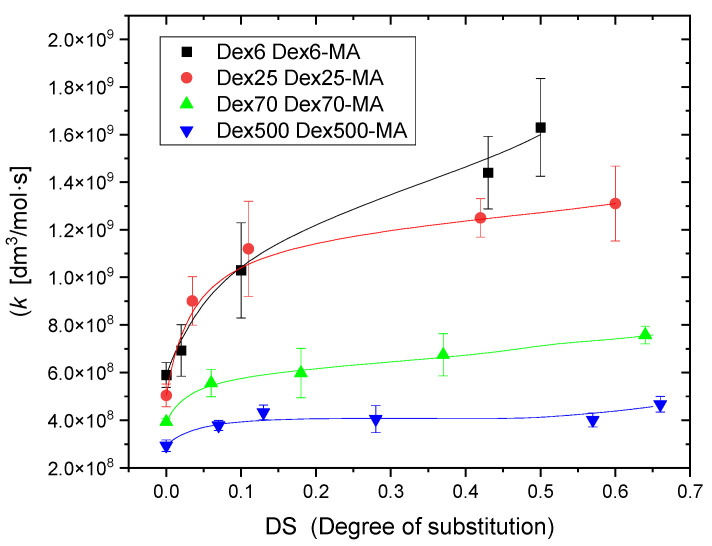
Rate constants of reaction of hydroxyl radicals with dextrans (DS = 0) and dextran methacrylates (Dex-MA) of various molecular weight, as a function of degree of substitution, determined by competition kinetics using pulse radiolysis. Experimental conditions as in Figure 2.

Each experiment was performed three times and average values were calculated. Summarized results presenting the rate constants for dextrans of various molecular weight, non-substituted and at various degrees of substitution, are shown in Figure 3.

It is clear, principally, that the rate constant of ^•^OH reaction with Dex-MA rises with the methacrylate content, which is particularly pronounced at low degree of substitution. Moreover, both for Dex and Dex-MA, the rate constant decreases with increasing molecular weight. These effects are discussed in Section 3.

Pulse radiolysis allows not only for the study of kinetics but also observing in time-resolved mode the spectra of transient species formed upon irradiation. While a complete study of spectral properties and time evolution for various pulse-irradiated Dex and Dex-MA samples is beyond the scope of our work, we have recorded selected spectra of reaction intermediates to support the discussion of the first steps of reaction mechanisms (see Section 3).

**Figure 4:**
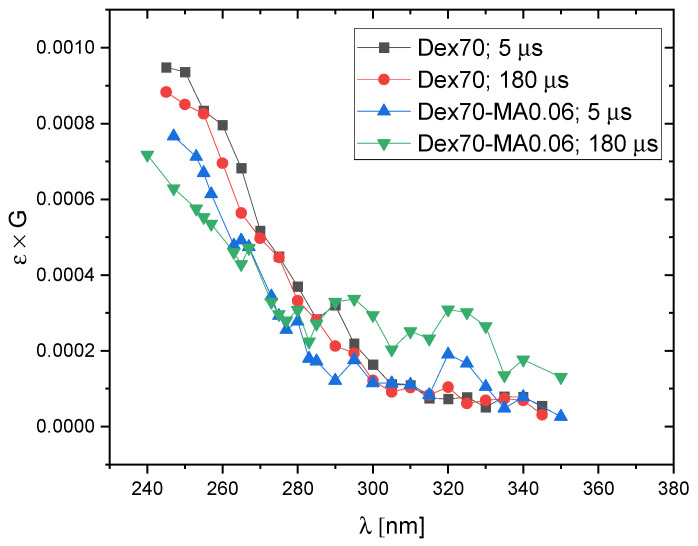
Pulse radiolysis of N_2_O-saturated aqueous solutions of Dex70 and Dex70-MA0.06. Absorption spectra at 5 μs and 180 μs after the pulse (pulse duration 17 ns, dose per pulse 50 Gy, polymer concentration 20 mM).

**Figure 5:**
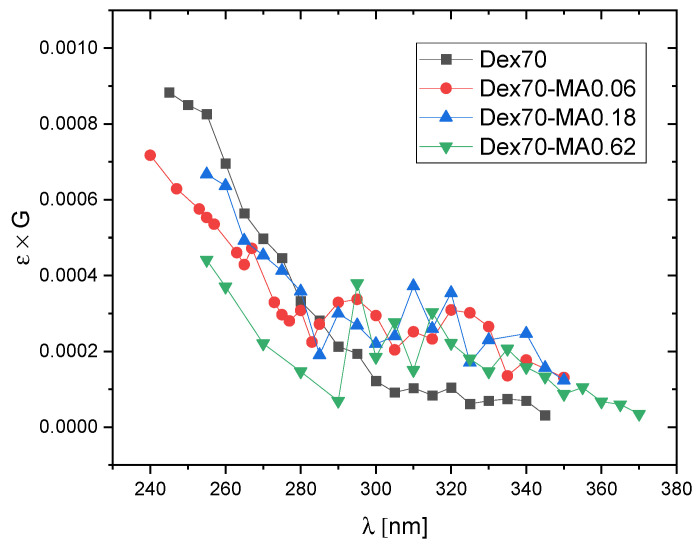
Pulse radiolysis of N_2_O-saturated aqueous solutions of Dex70, Dex70-MA0.06, Dex70-MA0.18 and Dex70-MA0.62. Absorption spectra at 180 μs after the pulse (pulse duration 17 ns, dose per pulse 50 Gy, polymer concentration 20 mM).

In order to compare the dominating products of ^•^OH attack on Dex and Dex-MA, transient spectra of radiolysis products observed at 5 μs and 180 μs after electron pulse in N_2_O-saturated solutions of pure dextran and dextran methacrylate of relatively low degree of substitution (DS = 0.06) are shown in Figure 4. It was also of interest to see if increasing DS has an effect on the observed spectra. In Figure 5, spectra recorded at 180 μs after the pulse for Dex-MA of various DS are compared.

### 2.3. Reactivity of Hydrated Electrons with Dextran and Dextran Methacrylate

Evaluation of rate constants of dextran and Dex-MA reactions with hydrated electrons were conducted by a direct observation of $e_{\mathrm{aq}}^{-}$ absorption decay at 720 nm. In these experiments, and also in those aimed at observations of transient spectra, ^•^OH radicals were eliminated by adding 0.2 M of tert-BuOH (reaction (5)) [13]. All experiments were conducted under argon atmosphere to deaerate environment of reaction, considering that $e_{\mathrm{aq}}^{-}$ reacts and decays rapidly in reaction with oxygen.

(5) $${}^{•}\mathrm{OH}+\mathrm{tertBuOH}\;\to {\;\mathrm{OH}}^{-}+{\;\mathrm{tertBuOH}}^{\cdot }$$

**Figure 6:**
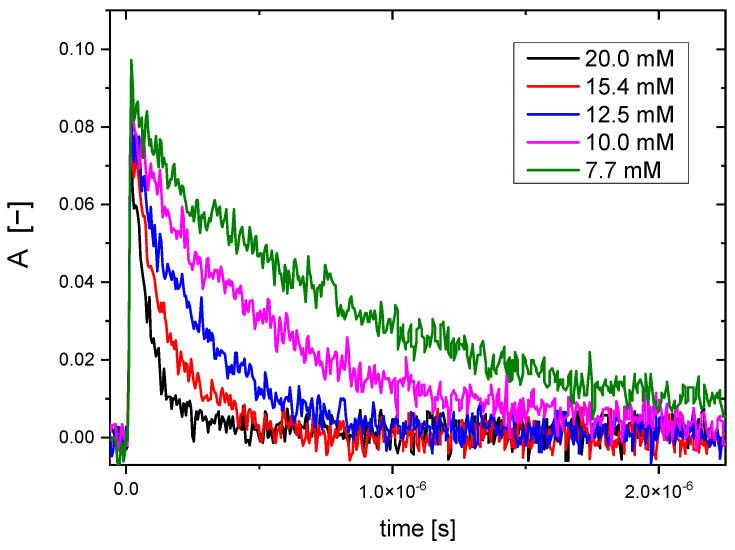
Pulse radiolysis of Ar-saturated aqueous solutions of Dex25-MA0.15 (polymer concentrations given in the graph) containing 0.2 M t-BuOH. Kinetic traces at 720 nm. Pulse duration 7 ns, dose per pulse 15 Gy.

**Figure 7:**
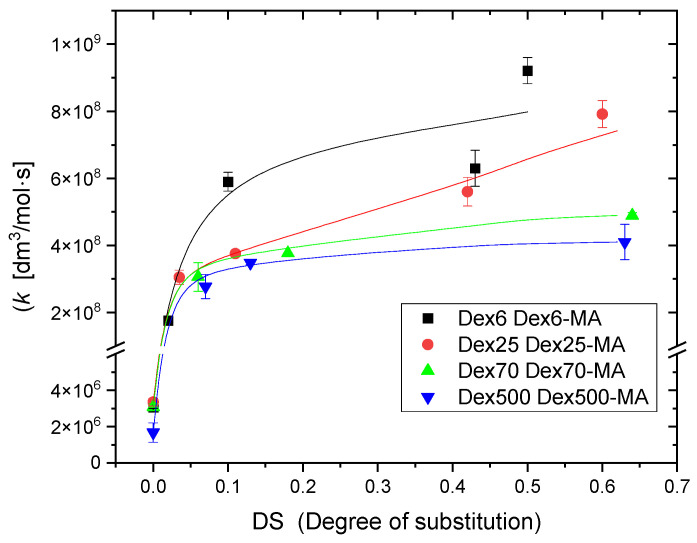
Rate constants of reaction of hydrated electrons with dextrans (DS = 0) and dextran methacrylates (Dex-MA) of various molecular weight, as a function of degree of substitution, determined by pulse radiolysis.

Electron decay in the presence of Dex is only moderately faster than in neat water, while Dex-MA strongly influences the $e_{\mathrm{aq}}^{-}$ lifetime. Exemplary decay traces of hydrated electron in the presence of various concentrations of Dex25-MA0.15 are shown in Figure 6. The decay can be described by pseudo-first-order kinetics. Based on the dependence of first-order rate constant on polymer concentration, second-order rate constants of reaction of $e_{\mathrm{aq}}^{-}$ with Dex and Dex-MA were calculated. These data are presented for different molecular weight as a function of DS (Figure 7).

**Figure 8:**
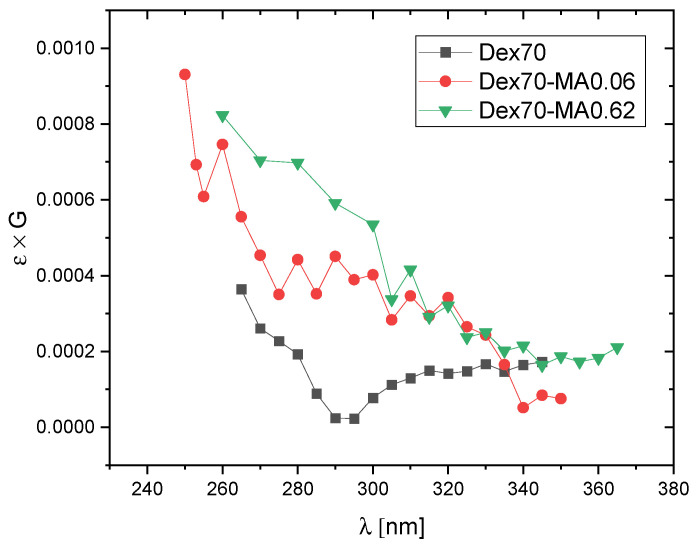
Pulse radiolysis of Ar-saturated aqueous solutions of Dex70, Dex70-MA0.06 and Dex70-MA0.62 containing 0.2 M t-BuOH. Absorption spectra at 1 μs after the pulse (pulse duration 17 ns, dose per pulse 50 Gy, polymer concentration 20 mM).

**Figure 9:**
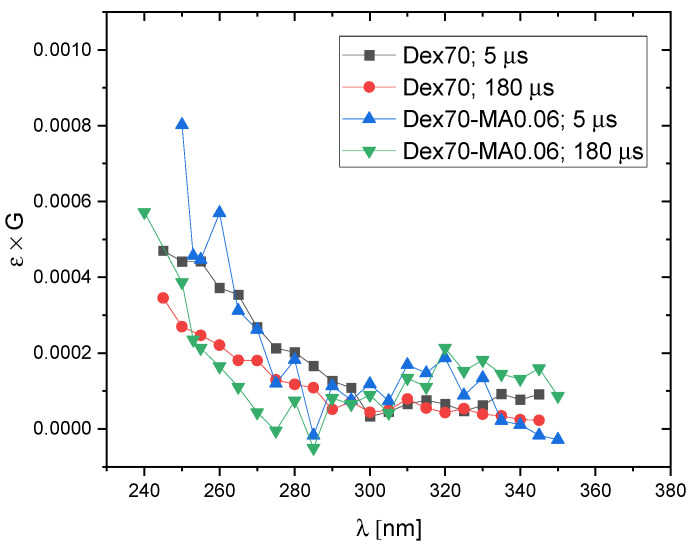
Pulse radiolysis of Ar-saturated aqueous solutions of Dex70 and Dex70-MA0.06 containing 0.2 M t-BuOH. Absorption spectra at 5 μs and 180 μs after the pulse (pulse duration 17 ns, dose per pulse 50 Gy, polymer concentration 20 mM).

Spectra shown in Figure 8 also demonstrate the pronounced difference of hydrated electron reactivity with unsubstituted dextran and Dex-MA. We can see that for pure Dex at 1 μs after the pulse the tail of the $e_{\mathrm{aq}}^{-}$ spectrum is clearly visible at λ > 300 nm with only weak absorbance below 300 nm, while the spectra for Dex-MA are very different in shape, showing stronger absorbance in the low wavelength region. Figure 9 illustrates the effect of methacrylate group on the spectra of transient products of hydrated electron with Dex and Dex-MA.

**Figure 10:**
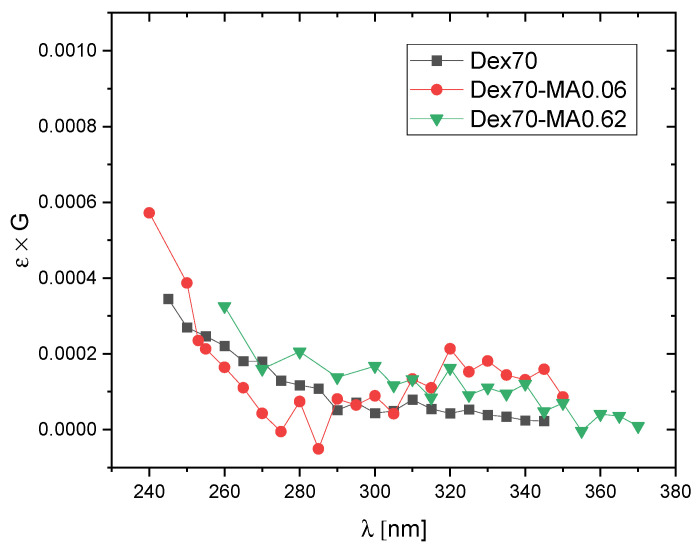
Pulse radiolysis of Ar-saturated aqueous solutions of Dex70, Dex70-MA0.06 and Dex70-MA0.62 containing 0.2 M t-BuOH. Absorption spectra at 180 μs after the pulse (pulse duration 17 ns, dose per pulse 50 Gy, polymer concentration 20 mM).

In order to trace the influence of degree of substitution on the structure and yield of hydrated electron reaction products with Dex-MA, transient spectra for substrates of various DS are presented in Figure 10.

**Figure 11:**
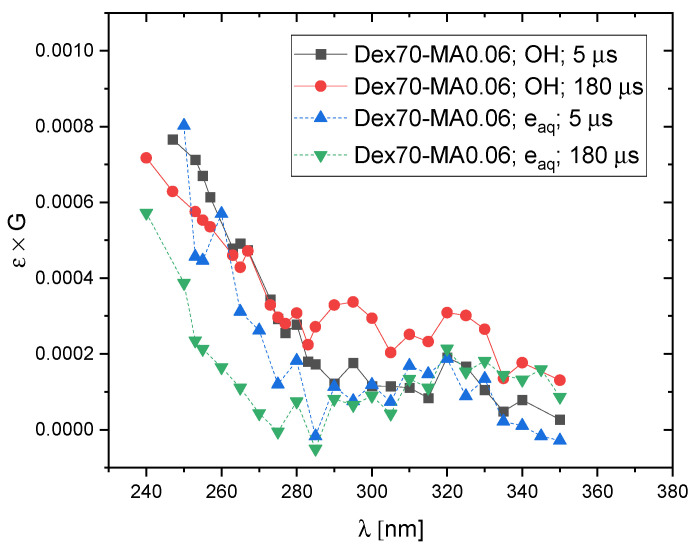
Pulse radiolysis of aqueous solutions of Dex70-MA0.06. Overlay of absorption spectra at 5 μs and 180 μs after the pulse (pulse duration 17 ns, dose per pulse 50 Gy, polymer concentration 20 mM) obtained in N_2_O-saturated solutions and in Ar-saturated solutions containing 0.2 M t-BuOH.

Finally, to illustrate the structural similarity of propagation-inducing radicals formed by ^•^OH–and $e_{\mathrm{aq}}^{-}$—initiated reactions in Dex-MA, spectra recorded for both these cases are presented in Figure 11.

## 3. Discussion

### 3.1. General Remarks

While this study is manly focused on the kinetics of ^•^OH and $e_{\mathrm{aq}}^{-}$ reactions with Dex and Dex-MA, besides discussing the kinetics-related results we also consider some spectral data collected in our experiments; these two approaches allow us to present some general remarks regarding the mechanism of main reactions taking place in the studied system.

Hydroxyl radicals are known to react with non-modified carbohydrates by hydrogen abstraction, thus generating C-centered hydroxyalkyl-type radicals. Selectivity of the ^•^OH attack on sugars is low, thus one may assume that these radicals are generated randomly at various positions. The rate constants of this reaction for low-molecular-weight sugars is in the order of *k* = 2 × 10^9^ dm^3^ mol^−1^ s^−1^ ([35], see above). As it is usually observed both for synthetic and natural polymers, the rate constants of their reactions with ^•^OH radicals in dilute aqueous solutions are typically lower than those for corresponding low-molecular-weight analogues, which is at least in part caused by the change of reaction geometry (polymer coils separated by large “void” volumes of water vs. relatively homogeneously distributed small molecules; this influences the average diffusion distance of ^•^OH, which is to be crossed, to reach the macromolecule), lower mobility of monomer units when embedded into a long chain and also steric hindrance. It should be mentioned that, even within the dilute concentration regime, the rate constant of ^•^OH reaction with a polymer does depend both on its concentration and molecular weight. When defined in dm^3^ per second and mol of monomer units, this rate constants decreases with increasing molecular weight. The amplitude of this effect for flexible chains, such as poly(*N*-vinylpyrrolidone), may amount to reducing the rate constant by one order of magnitude when moving from single monomer unit to a polymer of ca. 100 kDa. For more detailed studies and discussions on these effects, see [46,47,48].

In marked contrast to ^•^OH radicals, reactivity of hydrated electrons towards simple unsubstituted sugars, including polysaccharides, is very low, the rate constants being below *k* = 5 × 10^6^ dm^3^ mol^−1^ s^−1^ [35], which in practical terms means that the presence of sugar does not noticeably increase the rate of $e_{\mathrm{aq}}^{-}$ decay in comparison to pure water. This is also the case of our pulse-radiolysis observations on Dex + $e_{\mathrm{aq}}^{-}$ reaction, where values not exceeding *k* = 2–3 × 10^6^ dm^3^ mol^−1^ s^−1^ have been estimated (Figure 7).

The presence of acrylate or methacrylate function changes the situation dramatically. Hydroxyl radicals are known to react with simple acrylates or methacrylates at a diffusion-controlled rates, by addition to the C=C bond. Exemplary rate constants are *k* = 1.2 × 10^10^ dm^3^ mol^−1^ s^−1^ for methyl methacrylate [35] and *k* = 1.5 × 10^10^ dm^3^ mol^−1^ s^−1^ for butyl acrylate [49]. Similarly, hydrated electrons have been demonstrated to react rapidly with low-molecular-weight acrylates and methacrylates (for instance, *k* = 1.6 × 10^10^ dm^3^ mol^−1^ s^−1^ for butyl acrylate [49], *k* = 1.7 × 10^10^ dm^3^ mol^−1^ s^−1^ for poly(ethylene glycol) diacrylate—PEGDA 700 [50]). These reactions are the basics of the initiation step in radiation-induced polymerization of acrylate and methacrylate monomers in aqueous media, both in solution and emulsion. Here, we follow the influence of the content of methacrylate groups (defined as DS) and also the average molecular weight on the kinetics of ^•^OH and $e_{\mathrm{aq}}^{-}$ reactions with dextran methacrylate. These data, supported by transient spectra and previous literature data on the reactions with low-molecular-weight methacrylates, allow us to indicate the main reactions taking place in the studied systems.

### 3.2. Reactivity of Hydroxyl Radicals with Dextran and Dextran Methacrylate

In the molecules of dextran methacrylate, the unsaturated carbon-carbon bonds of the methacrylate group are a favorable site for ^•^OH attack due to the high electron density and suitability for ^•^OH attachment reaction. This is reflected in the fact that the rate constant of ^•^OH reaction with methacrylate monomers is *k* = 1–2 × 10^10^ dm^3^ mol^−1^ s^−1^ (see above), i.e., one order of magnitude higher than with glucose, which can serve as a model of single dextran unit. The former value can be assumed as an upper limit of possible rate constant of ^•^OH radical and Dex-MA with increasing degree of substitution, at low molecular weight of the polymer.

With increasing degree of substitution of dextran monomer units with methacrylate groups, we expect the ratio of ^•^OH radicals undergoing addition to methacrylate vs. reacting by hydrogen abstraction from dextran backbone to increase as well. This effect is indeed observed in our kinetic data. Interestingly, in the range of low DS, this dependence seems to follow simple competition rules. Assuming, on the basis of the above-cited literature data, that the rate constant of ^•^OH reaction with methacrylate is ca. 10 times higher than with a sugar monomer unit, we shall expect that incorporating 10% (mol) of methacrylate into dextran would lead to equal participation of both reaction types in the reaction with ^•^OH, thus doubling the rate constant observed for pure dextran. The experimentally observed enhancement factors for DS = 0.1 are indeed close to this expected value, with the exception of Dex6 (which, being the most difficult to purify, could have contained some low-molecular-weight impurities increasing the observed rate constant for the non-modified sample). At higher DS values we observe a deviation from this simple competition rule. While we can see constantly increasing rate constant values with increase in DS, the effect becomes moderate and the values are lower than expected from simple competition. This can be caused by changes in chain conformation caused by the presence of high amount of added side groups, both by steric effects caused by the bulkiness of these groups and by the change in intramolecular interactions between chain segments, where methacrylate units may have the tendency to associate due to their more hydrophobic properties. These effect may influence both the volume occupied by macromolecules (thus changing the diffusion properties of the solution) and the accessibility of methacrylate groups for ^•^OH radicals within the polymer coils.

Another aspect worth discussing is the dependence of the rate constants on molecular weight, observed both for Dex and Dex-MA. The values of rate constant for ^•^OH attack on pure dextrans obtained here (Figure 3) are, as expected, somewhat lower than the value for glucose. Moreover, they decay with increasing molecular weight (from *k* = 9 × 10^8^ dm^3^ mol^−1^ s^−1^ for Dex6 down to *k* = 3 × 10^8^ dm^3^ mol^−1^ s^−1^ for Dex500, an expected trend that has been observed for other water-soluble polymers before [46,51,52,53]. The same tendency is evident from data shown in Figure 3 also for dextran methacrylates. For instance, at DS ≈ 0.5, the rate constants decrease from *k* = 1.6 × 10^9^ dm^3^ mol^−1^ s^−1^ for Dex6 methacrylate down to *k* = 4 × 10^8^ dm^3^ mol^−1^ s^−1^ for Dex500 methacrylate. Since the ^•^OH reaction with low-molecular-weight methacrylates is diffusion controlled, the presence of molecular weight dependence for Dex-MA clearly indicates that the reaction rate of ^•^OH with methacrylated dextran is controlled by diffusion as well, but here the diffusion is governed by the micro-heterogeneous geometry of dilute polymer solution, becoming more pronounced with increase in molecular weight.

The fate of carbon-centered radicals in unsubstituted polysaccharides formed as a result of ^•^OH attack in oxygen-free systems depends to some extent on their localization. Those located in the vicinity of glycosidic bonds may transform with breakage of these bonds, thus leading to chain scission and reduction of the average molecular weight. The latter effects of dextran radiolysis in dilute aqueous solutions have been known since the first studies on this topic were undertaken in 1950s [10]. Incorporation of methacrylate groups able to undergo cross-linking polymerization allows us to overcome the (usually unwanted) tendency of the polysaccharide to degrade under irradiation; cross-linking polymerization of Dex-MA is so efficient that 3D gels based on covalently cross-linked chain network are formed at irradiation doses as low as 200 Gy (J/kg) [29].

A proposed scheme of main reactions induced by ^•^OH reaction with the methacrylate group of Dex-MA is shown as Equations (6)–(8). Addition of the hydroxyl radical to the C=C bond (reaction (6)) predominantly creates an α-carboxyalkyl radical/**1**/. The latter can add to the double bond on the same of other Dex-MA molecule, inducing a chain reaction of propagation (reaction (7), structure/**2**/). Since the Dex-MA molecules involved have many MA groups available, this is actually cross-linking polymerization leading to the formation of 3D network. Moreover, besides addition, such radicals can undergo recombination (reaction (8)), either by disproportionation or by cross-linking, leading to products/**3**/and/**4**/or/**5**/, respectively. These two reactions of propagating radicals are expected to be the main radical termination modes in the studied system. Other reactions of minor importance (not shown) could be, e.g., radical transfer by H-abstraction. Of course, also the radicals formed, in minor quantities, by ^•^OH attack on the Dex backbone, could participate in recombination. They also can rearrange with breakage of the glycosidic bond, a reaction which may make the dominating network formation process somewhat less efficient. On the other hand, radicals generated upon glycosidic bond scission, located at the newly formed chain end, may also contribute to the network formation, or reduce degradation effect, if reacted with C=C double bond to attach the split chain to another macromolecule.

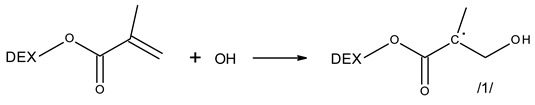
(6)

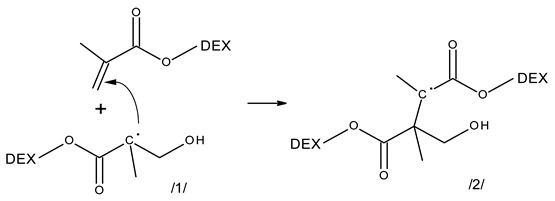
(7)

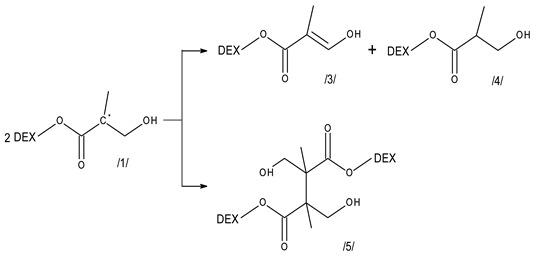
(8)

Radicals formed by ^•^OH addition to the C=C bond of the methacrylate group (predominantly structure/**1**/) are expected to yield broad spectra in UV rising towards low wavelength, but extending to λ > 350 nm [49,54,55]. In some cases (butyl acrylate, tri(propylene glycol) diacrylate) at short times a broad maximum at ca. 300 nm has been observed [49,55], and there is some general tendency for absorbance increase at the far-UV part of the spectrum [49,54]. While addition of this radical to the next methacrylate group and the following chain reaction of propagation should not affect the radical spectra (the structure of the propagating radical does not change), at the same time propagation depletes the concentration of methacrylate groups in the system, which may cause a decay in the basic absorbance compared to the “zero” time. Moreover, it should be mentioned that also the final, stable products, such as the unsaturated structures/**3**/, expected to absorb, depending on pH, between 250 and 270 nm, can contribute to some extent to the observed spectra, especially at the longest times studied here, and beyond. Therefore, while there is no much doubt as to the character of main reactions initiated in Dex-MA system by ^•^OH radicals, featureless character of the spectra, overlay of the (similar) spectra resulting from H abstraction from the sugar backbone and from ^•^OH addition to the C=C bond of the methacrylate group and parallel occurrence of several reactions influencing absorption makes it difficult to extract more detailed information from the spectral evolution of this system.

These expectations are in line with the spectra presented in Figure 4 and Figure 5. In Figure 4 we see that the spectra for non-substituted Dex70 are totally featureless, as expected for sugar radicals formed by H-abstraction by hydroxyl radicals, while the spectra for Dex70-MA0.06, in particular at 180 μs after the pulse, show a weak side absorption band at ca. 290–340 nm characteristic for methacrylate adduct radicals. The amplitude of this band doesn’t seem to increase strongly for higher DS values (Figure 5) which indicates that, due to much higher rate of ^•^OH addition when compared to H-abstraction, already at DS = 0.06 significant part of hydroxyl radicals react according to the former mechanism.

### 3.3. Reactivity of Hydrated Electrons with Dextran and Dextran Methacrylate

Compared to ^•^OH-induced reactions, even more dramatic effect is exerted by the presence of methacrylate groups on the reactivity of dextran with hydrated electrons (Figure 7). We should bear in mind that non-substituted dextrans, in practical terms, does not react with $e_{\mathrm{aq}}^{-}$, therefore the presence of even small amount of methacrylate groups (DS of 0.02–0.07) has a very pronounced effect on reactivity. Further increase of DS does lead to some rise in the rate constant, but, probably due to the similar conformation effects as discussed above, the effect is definitely less than proportional to the methacrylate content.

The effect of Dex-MA molecular weight on the rate constant with $e_{\mathrm{aq}}^{-}$ is similar as in the case of ^•^OH reactions; the rate constant (calculated in dm^3^ per second and mol of monomer units) decreases with polymer chain length. Again, several factors can be invoked here—lower mobility of monomer units when embedded in long chains, change in reaction geometry (more heterogeneous system with large void pools of water between long chains, necessitating longer $e_{\mathrm{aq}}^{-}$ diffusion to reach a reaction site) and, possibly, also steric hindrance which may be expected due to potential clustering of hydrophobic methacrylate groups on long chains.

Reactions of hydrated electrons with acrylates and methacrylates in water have been the subject of several studies. While the findings and interpretations presented in these works differ in some details (see below), a general scheme of the mechanism is well established and is expected to operate also for Dex-MA. Hydrated electrons add to acrylate and methacrylate functions (reaction (9)), forming short-lived radical anions (structure/**6**/). The latter is expected to undergo protonation reactions. One of them is reversible and thus pH-dependent, it leads to the ketyl-type radical/**7**/. Irreversible, relatively slow protonation results in the formation of α-carboxyalkyl radicals/**8**/. It is not clear whether the ketyl-type radicals can re-arrange to the of α-carboxyalkyl ones; such process has been postulated [54], but not observed in the detailed study of Mehnert et al. [56].
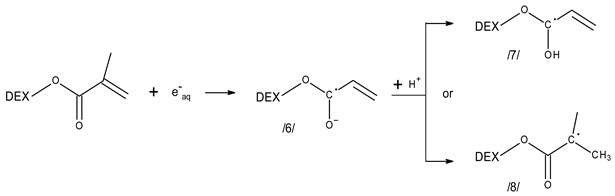
(9)

Since our study has been made in neutral solution, the yield of ketyl-type radicals/**7**/is expected to be moderate, thus we believe that most of the radical anions are transformed into the of α-carboxyalkyl radicals/**8**/. From the point of view of radiation-induced polymerization, precise differentiation between both protonation pathways is not of prime importance, since both types of resulting radicals are able to initiate polymerization.

The radical anion is expected to have an absorption spectrum in the UV region, with a maximum at ca. 250–280 nm. Its protonation leads to the two above-mentioned radicals which have been demonstrated on similar acrylate structures to have a spectrum rising monotonously towards low wavelength for the ketyl-type radical or/and a relatively weak spectrum with a broad maximum between 300 and 350 nm for the of α-carboxyalkyl radicals.

We see that after completion of $e_{\mathrm{aq}}^{-}$ reaction with Dex-MA and at least partial (at 5 μs) or complete (at 180 μs) protonation of the radical-anions the spectra of products derived from Dex-MA in fact show a broad band in the 300–350 nm region, of more pronounced absorbance than non-substituted Dex. While the kinetics of protonation hasn’t been studied here, it can be estimated that after 180 μs after the pulse protonation is expected to be complete and only few of the so formed radicals have already reacted by addition to other methacrylate groups (for estimation method in pulse-induced polymerization, see e.g., [57,58]), thus it is expected that most of the radicals present at that time would be the protonation products/**8**/and/**7**/, their spectra being in line with expectations based on earlier studies on radiolysis of acrylates and methacrylates. Those radicals are anticipated to participate in propagation and cross-linking reactions, similarly to transient products of ^•^OH radical reactions with methacrylate moiety, contributing to network formation.

Spectra recorded at 180 μs after the pulse (Figure 10) show that in the 300–350 nm range absorbance in Dex-MA solutions is stronger than in case of unsubstituted Dex. However, similarly as in the case of ^•^OH-induced reactions, there is no much difference in absorbance between Dex70-MA0.06 and Dex70-MA0.62; in both cases almost all $e_{\mathrm{aq}}^{-}$ have reacted with methacrylate group and the product concentration is controlled mainly by the quantity of hydrated electrons, directly dependent on the dose, which was the same for both samples.

Since we expect of α-carboxyalkyl radicals/**8**/to be the dominating protonation product of electron adduct in our system, the spectrum observed due to its formation should be similar to the spectrum of ^•^OH addition product/**1**/. In fact, the spectra observed in both cases at 5 μs and 180 μs after the pulse are of similar shape (Figure 11). Both these radicals can add to the C=C bond of another methacrylate group thus initiating propagation. Even if we assume that some single acts of propagation might have already taken place within the studied timeframe (180 μs), the propagating radicals in both cases would have the same structure and thus the same spectral properties.

## 4. Materials and Methods

### 4.1. Materials

Dextrans (from *Leuconostoc* spp., *Mr* = 6–500 kDa), dimethyl sulfoxide (DMSO, anhydrous, ≥99.9%), glycidyl methacrylate (GMA, 97%, stabilized by 0.005% hydroquinone monomethylether), 4–(*N*,*N*-dimethylamino)pyridine (DMAP) and cellulose dialysis membrane tubes (MWCO 14 kDa) were purchased from Sigma Aldrich (Poznan, Poland). Hydrochloric acid (HCl, 36–38%) and potassium thiocyanate (KSCN) were acquired from Chempur (Piekary Slaskie Poland). Tert-butanol Emplura (tert-BuOH, 99%) was purchased from Merck KGaH Group (Darmstadt, Germany). Dialysis membranes from regenerated cellulose with MWCO 3.5 kDa and 13 kDa were delivered by SERVA Electrophoresis GmbH (Heidelberg, Germany). Gases of high purity (99.999%): nitrous oxide (N_2_O) and argon (Ar), were purchased from Linde Group and Air Products, respectively. All reagents, with the exception of Dex, were used as received. Ultra-purified water (>18 MΩ·cm) was obtained using MicroPure system (TKA, Thermo Scientific, Waltham, MA, USA).

### 4.2. Dextran Purification and Synthesis of Dextran Methacrylate

To reduce the unfavorable influence of presence of oligosaccharides and lower-molecular-weight fractions, and also to remove potential low-molecular-weight impurities, all dextrans used in pulse radiolysis experiments were prepurified by dialysis against pure water. The molecular weight cutoff (MWCO) of the cellulose membrane was selected according to Dex initial molecular weight.

A series of Dex-MA of different initial molecular weight (nominal *Mr* of original dextran substrate = 6, 25, 70 and 500 kDa) and a wide range of degree of substitution (DS) was synthesized using the procedure of van Dijk-Wotthuis [59]. The synthesis and characterization methods of Dex-MA are described in detail elsewhere [29]. In short, Dex-MA batches were synthesized in anhydrous dimethyl sulfoxide used as a solvent, in the presence of a catalysts, 4–(*N*,*N*-dimethylamino)pyridine with glycidyl methacrylate added in molar ratio with respect to dextran, determined for expected DS. The synthesis proceeded for 48 h under ambient gas atmosphere, and the reaction was terminated with HCl, equimolar to the catalyst. A prolonged dialysis against water was employed to purify the product prior its lyophilization. Synthesized dextran derivatives were characterized by proton nuclear magnetic resonance spectroscopy (^1^H–NMR, Bruker Avance II 700 MHz UltraShield Plus, Karlsruhe, Germany) in order to determine degree of methacrylate substitution. The intended DS of Dex-MA series was 0.1–0.7, which ensures complete solubility in water. The actual values of DS obtained in synthesis of Dex-MA series are shown in Table 1. Dex-MA samples are marked in the text by the average molecular weight of the parent dextran (e.g., 500 for 500 kDa) and their DS (e.g., 0.66); samples of these parameters is coded Dex500-MA0.66.

### 4.3. Pulse Radiolysis

A nanosecond pulse radiolysis system with time-resolved spectroscopic detection based on a 6 MeV linear accelerator ELU–6 Linac (Elektronika, Moscow, Russia) was used in all experiments. The pulse duration was 7 or 17 ns, depending on the type of experiment, resulting in doses per pulse of ca. 15 Gy and 50 Gy, respectively. N_2_O-saturated aqueous solution of potassium thiocyanate (10 mM) was applied for dosimetric measurements. The dose uncertainty was up to 10%. The detection system consisted of a 50 W Xenon lamp, monochromator (Spectra Pro 275, Princeton Instruments, New Jersey, NJ, USA), photomultiplier (Hamamatsu, Hamamatsu, Japan), and digital oscilloscope (Tektronix TDS540, Salem, OR, USA). The electron beam and analyzing light were set on a horizontal plane, perpendicularly to each other. A quartz cuvette of 1 cm optical path length was used. A water filter was applied to remove the IR part of lamp spectrum. For recording the thiocyanide radical anion (SCN)_2_^•−^ absorbance at λ = 480 nm in the competition kinetic experiments and the decay of hydrated electron at λ = 720 nm a glass filter was additionally used to block the UV part of lamp spectrum. Further details of the setup are described elsewhere [60].

All experiments were performed in neutral aqueous solutions. Polymer solutions were prepared at least one day before radiolysis to ensure complete dissolution of macromolecular samples. Polymer concentrations are expressed in mol of monomer units per dm^3^. As a consequence, the rate constants are reported here in dm^3^ per second and mol of monomer units.

## 5. Conclusions

The presence of methacrylate groups on dextran molecules, allowing for fast and efficient ^•^OH addition and $e_{\mathrm{aq}}^{-}$ addition reactions upon irradiation in aqueous solution, are beneficial from the point of view of radiation synthesis of Dex-MA hydrogels. What’s more, this effective action manifests itself already for low degrees of dextran substitution with methacrylate groups (even below DS = 0.1), which is in line with previous experimental observations on very efficient hydrogel formation taking place already for low DS and low doses of radiation. Transient products of ^•^OH and $e_{\mathrm{aq}}^{-}$ reactions with methacrylate double bonds and carbonyl groups, that is, radicals located at the side group of dextran derivative, undergo polymerization and recombination, thus advancing cross-linking. High cross-linking polymerization rate and efficiency at low DS values is also advantageous for the potential biomedical applications of radiation-synthesized Dex-MA hydrogels since it allows us to avoid potential biocompatibility problems, which were encountered only for gels of DS exceeding 0.8.

## Figures and Tables

**Table 1:** Specification of synthesized Dex-MA samples.

Initial Molecular Weight of Dextran Substrates (kDa)	Series of Dex-MA	Determined DS ^1^
6	Dex6-MA	0.02, 0.11, 0.43, 0.50
25	Dex25-MA	0.04, 0.11, 0.42, 0.60
70	Dex70-MA	0.06, 0.18, 0.37, 0.64
500	Dex500-MA	0.07, 0.13, 0.28, 0.57, 0.66

^1^ DS (degree of substitution)—average number of methacrylate groups per D-glucopyranose residue.

## Data Availability

Data are available from authors on reasonable request.
